# Trends in US President’s Malaria Initiative-funded indoor residual spray coverage and insecticide choice in sub-Saharan Africa (2008–2015): urgent need for affordable, long-lasting insecticides

**DOI:** 10.1186/s12936-016-1201-1

**Published:** 2016-03-08

**Authors:** Richard M. Oxborough

**Affiliations:** Richard Oxborough Consultancy, London, UK

## Abstract

This article reports the changing pattern of US President’s Malaria Initiative-funded IRS in sub-Saharan Africa between 2008 and 2015. IRS coverage in sub-Saharan Africa increased from <2 % of the at-risk population in 2005, to 11 % or 78 million people in 2010, mainly as a result of increased funding from PMI. The scaling up of IRS coverage in sub-Saharan Africa has been successful in several epidemiological settings and contributed to reduced malaria transmission rates. However, the spread and intensification of pyrethroid resistance in malaria vectors led many control programmes to spray alternative insecticides. Between 2009 and 2013, pyrethroid spraying decreased from 87 % (13/15) of PMI-funded countries conducting IRS to 44 % (7/16), while bendiocarb use increased from 7 % (1/15) to 56 % (9/16). Long-lasting pirimiphos-methyl CS received WHOPES recommendation in 2013 and was scheduled to be sprayed in 85 % (11/13) of PMI-funded countries conducting IRS in 2015. The gradual replacement of relatively inexpensive pyrethroids, firstly with bendiocarb (carbamate) and subsequently with pirimiphos methyl CS (organophosphate), has contributed to the downscaling of most PMI-funded IRS programmes. Overall, there was a 53 % decrease in the number of structures sprayed between years of peak coverage and 2015, down from 9.04 million to 4.26 million structures. Sizeable reductions in the number of structures sprayed were reported in Madagascar (56 %, 576,320–254,986), Senegal (64 %, 306,916–111,201), Tanzania (68 %, 1,224,095–389,714) and Zambia (63 %, 1,300,000–482,077), while in Angola, Liberia and Malawi PMI-funded spraying was suspended. The most commonly cited reason was increased cost of pesticides, as vector resistance necessitated switching from pyrethroids to organophosphates. There are worrying preliminary reports of malaria resurgence following IRS withdrawal in parts of Benin, Tanzania and Uganda. The increase in malaria cases following the end of the Global Malaria Eradication Programme in 1969 highlights the fragility of such gains when control efforts are weakened. At present there are several countries reliant on organophosphates and carbamates for IRS, and increasing incipient resistance is a serious threat that could result in IRS no longer being viable. A portfolio of new cost-effective insecticides with different modes of action is urgently needed.

## Background

Indoor residual spraying (IRS) of insecticides has produced profound changes in malaria burden in a range of settings, including the elimination of malaria through indoor spraying of DDT (in combination with environmental management, improved housing and treatment) during the Global Malaria Eradication Programme (GMEP, 1955–1969) in the USA, Europe, parts of the Soviet Union, Israel, Lebanon, Syria, Japan, and Taiwan [1]. While IRS in Africa was largely overlooked during the GMEP, sustained IRS programmes have been successfully staged in South Africa, Zambia, Namibia, Swaziland, Zimbabwe and Botswana for several decades since the 1940s and have achieved sizeable reductions in vector populations and malaria incidence [2, 3]. IRS is applicable in many epidemiological settings, including hyperendemic areas of sub-Saharan Africa, provided that policy and programming decisions take into account the operational and resource feasibility [4]. In 2006, the World Health Organization (WHO) reaffirmed the importance of IRS as a primary intervention for reducing or interrupting malaria transmission [5]. The US President’s Malaria Initiative (PMI) was launched in 2005, initially as a 5 year, $1.2 billion programme to rapidly scale up malaria prevention and treatment interventions and reduce malaria-related mortality by 50 % in 15 high-burden countries in sub-Saharan Africa [6]. Priority was given to the scale up of four proven interventions; namely long-lasting insecticidal nets (LLINs), IRS, intermittent preventive treatment in pregnancy (IPTp), and prompt diagnosis with rapid diagnostic tests (RDTs) and treatment with artemisinin-based combination therapy (ACT) [6]. IRS coverage in sub-Saharan Africa increased substantially from <2 % of the at-risk population protected in 2005, to 11 % or 78 million people in 2010 [7]. Increased IRS coverage was successful in a range of diverse epidemiological settings, with substantial reductions for all cause child mortality, malaria parasite prevalence and entomological indicators recorded in several countries including parts of Equatorial Guinea (not a PMI-supported country) [8], Benin [9], Kenya [10], Malawi [11], and Tanzania [12].

The duration of residual efficacy and cost-effectiveness of IRS insecticides are of key importance to the sustainability of IRS programmes. A major challenge facing IRS programmes is to sustain such gains in the face of technical problems, such as vector resistance to insecticides, lack of affordable alternative insecticides and limited resources for annual renewal of each intervention. In this article the changing pattern of PMI-supported IRS in sub-Saharan Africa between 2008 and 2015 is documented and the technical challenges being faced are examined. A limitation is that data was not included for IRS conducted through other funding sources such as UK Department for International Development (DFID), The Global Fund to Fight AIDS, Tuberculosis and Malaria, private companies and national Governments.

## Insecticide resistance and scaling down of IRS

There are four classes of insecticide recommended for IRS for malaria control and prevention, each with various attributes (Table 1). Global use of vector control insecticides was dominated by pyrethroids in terms of surface area covered (81 % of total) between 2000 and 2009, with the upsurge in use of pyrethroid IRS partly as a result of PMI-funded spraying in Africa [13]. The scaling up of pyrethroid IRS in sub-Saharan Africa between 2006 and 2010 was attainable as pyrethroid insecticides were inexpensive and had a relatively long residual action [14, 15]. However, increased coverage of pyrethroid IRS, often in parallel with pyrethroid LLIN distribution and agricultural use, has led to the spread and intensification of pyrethroid resistance across most of malaria endemic sub-Saharan Africa [16, 17]. WHO has since recommended that pyrethroid IRS should not be used for IRS in areas of moderate to high LLIN usage, in an attempt to preserve the effectiveness of pyrethroid LLINs [18].

**Table 1:** Attributes of insecticide formulations commonly used for IRS for malaria control and prevention

^a^Frequency of vector resistance recorded in sub-Saharan Africa [56]

^b^Related to the volume of formulation required, packaging, ease of shipping and disposal or recycling requirements

Despite the Stockholm Convention on Persistent Organic Pollutants stipulating that, ‘countries using DDT should eliminate the use of DDT over time and switch to alternative insecticides’, the use of DDT for malaria control has been allowed to continue under exemption [19, 20]. DDT has a long residual action of more than 6 months and is relatively inexpensive, with a cost similar to pyrethroids [14, 15]. However, there is high frequency of mutations in the *knockdown resistance* gene (*kdr*) across sub-Saharan Africa, conferring cross-resistance to DDT and pyrethroid insecticides [17]; in addition there is political resistance, with several countries reluctant to register or utilize DDT due to perceived environmental or export concerns [21, 22].

The carbamate bendiocarb is highly effective, but has a short residual duration, meaning that multiple spray cycles may be required for optimal protection in areas of prolonged transmission [23, 24]. Propoxur is a carbamate insecticide that is currently used for IRS in Ethiopia, but is not utilized through the PMI as the product specifications are not listed under the Joint FAO/WHO Meeting on Pesticide Specifications (JMPS) [25].

Pirimiphos-methyl (p-methyl) emulsifiable concentrate (EC), an organophosphate insecticide that was developed in the 1970s, shows high toxicity to *Anopheles* mosquitoes but has infrequently been utilized despite having WHO recommendation for IRS [15, 26]. Between 2009 and 2012, p-methyl EC was sprayed in Malawi, Benin and Zambia but was subsequently replaced due to the prohibitive expense and short residual activity [27, 28]. In 2013, a new formulation of p-methyl capsule suspension (CS) was recommended by WHO for IRS, with experimental hut trials demonstrating vastly improved residual duration of up to 12 months [29–31].

The spread and intensification of pyrethroid resistance in malaria vectors has led to policy changes regarding insecticide choice for IRS programmes. There is limited epidemiological evidence of pyrethroid IRS or LLIN failure, possibly due to a shortage of suitable data collection or alternatively pyrethroid insecticides may successfully kill older, less resistant infective mosquitoes and also provide some protection through repellency and the physical barrier effect of LLINs [32]. WHO cylinder bioassays have demonstrated an increased frequency of resistant malaria vectors and this has led many control programmes to stop spraying pyrethroids in favour of alternative insecticides [28, 33].

**Fig. 1:**
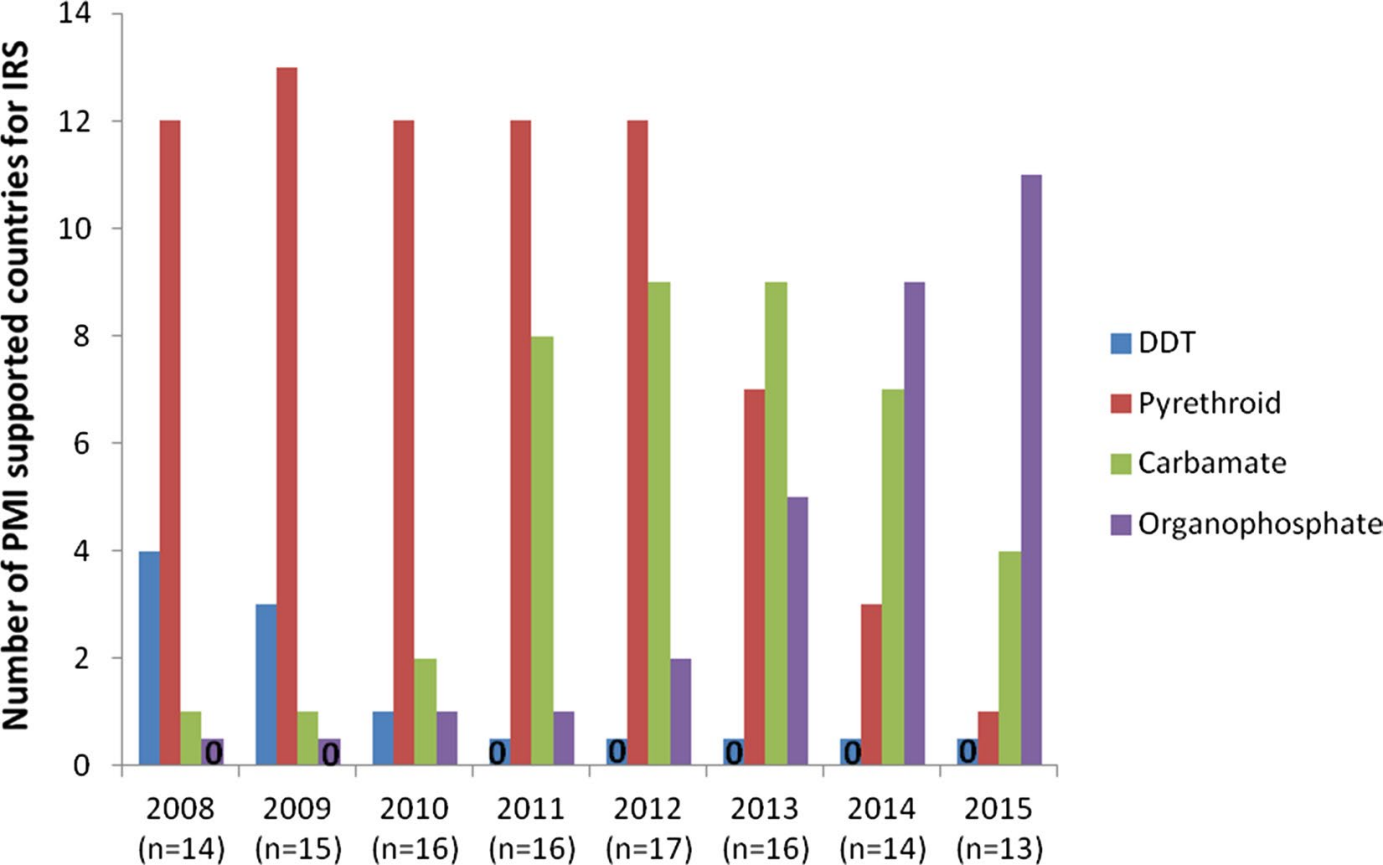
PMI-funded insecticide use for IRS between 2008 and 2015 (based on [14, 28] and 2015 PMI malaria operational plans)

In 2009, 87 % (13/15) of PMI-funded countries with IRS programmes sprayed pyrethroid IRS (Fig. 1). This proportion decreased annually, with only 44 % (7/16) of countries having sprayed pyrethroids in 2013, while carbamate use increased during the same period from 7 % (1/15) in 2009 to 56 % (9/16) in 2013 (Fig. 1). Bendiocarb was sprayed despite being relatively expensive and having a limited residual duration, as there were few viable alternatives (namely DDT and organophosphates). P-methyl EC was rarely utilized and was sprayed in <10 % of countries between 2009 and 2012. Following the development of a long-lasting formulation of p-methyl CS came a dramatic shift in usage, with 85 % (11/13) of PMI-supported countries spraying p-methyl CS in 2015 (Fig. 1), compared with 31 % (5/16) in the first year of production in 2013. The gradual replacement of relatively inexpensive pyrethroids firstly with bendiocarb (carbamate) and subsequently with p-methyl CS (organophosphate) has also led to substantial changes in IRS spray coverage. The cost of a pyrethroid (Icon™ lambdacyhalothrin capsule suspension (CS) 10 % ai, Syngenta) sachet is estimated to be $5, compared with $12 for bendiocarb (Ficam™ WP 80 % ai, Bayer CropScience), $20 for a container of p-methyl EC (Actellic™ EC, 50 % ai, Syngenta) and $23.50 for p-methyl CS (Actellic™ CS, 30 % ai, Syngenta), all with equivalent quantity of active ingredient to spray 250 m^2^ at WHO recommended target dosages [34]. It should be noted that there are additional insecticide cost drivers such as shipping, disposal of insecticide packaging, and environmental precautions, which vary according to formulation. For example, DDT WP may be the cheapest formulation at $2 but the additional environmental precautions and testing needed can increase overall costs considerably [35]. The type of insecticide formulation purchased is one of the most important cost drivers for spray programmes. In 2014, insecticides were the second largest cost category across PMI IRS programmes (per 100 m^2^) and accounted for 28 % of the total unit cost (across all countries regardless of insecticide formulation sprayed) [36]. For the few countries where pyrethroids were sprayed, insecticide accounted for only 6 % of all costs compared with 37 % where organophosphates were used [36]. However, it should be noted that there was considerable variation between countries in spray operations costs.

**Fig. 2:**
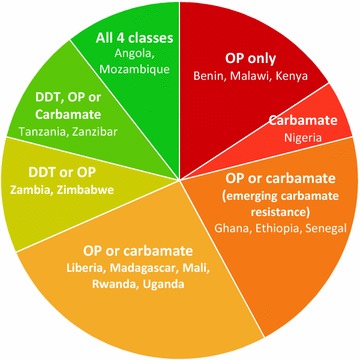
Insecticide classes considered as a viable option for IRS in 2015 (i.e., susceptible malaria vectors present in areas undergoing IRS). An insecticide was considered as not a viable option for IRS if it had previously been replaced due to vector resistance, was not registered for use, or susceptibility tests demonstrated widespread resistance (based on PMI operational plans FY2015 and [57])

The proportion of PMI-supported countries spraying bendiocarb increased greatly between 2011 and 2013 (Fig. 1), yet the number of structures sprayed remained high in most countries despite the increased cost compared to pyrethroids (12 countries reached peak numbers of structures sprayed between 2011 and 2013 (Table 2). This increase in cost was exacerbated by subsequent replacement with more expensive p-methyl CS, which, by 2015, had become the insecticide of choice for the majority of PMI-funded spray programmes (Fig. 1). Despite the increased cost, p-methyl CS was preferred to bendiocarb WP due to the extended duration of action (offsetting some of the added cost) and also in response to increasing reports of bendiocarb resistance in countries including Malawi, Benin, Ghana, Ethiopia and Senegal (Fig. 2) [4, 37, 38].

**Table 2:** Decrease in number of house structures sprayed with insecticide for malaria prevention between year of peak coverage and 2015 (data taken from PMI national malaria operational plans 2010–2016) [21, 22, 28, 37–45, 74–80]

Country	Year of peak coverage	Structures sprayed in year of peak coverage	Structures sprayed in 2015	% Reduction in structures sprayed
Angola	2011	145,264	0	100
Benin	2014	254,072	252,706	1
Ethiopia	2011	858,657	670,303	22
Ghana	2012	371,362	231,345	38
Kenya	2008	764,050	0	100
Liberia	2012	96,901	0	100
Madagascar	2010	576,320	254,986	56
Malawi	2010	97,329	0	100
Mali	2013	228,985	131,894	42
Mozambique	2011	660,064	440,579	33
Nigeria	2013	62,592	0	100
Rwanda	2011	358,804	213,271	41
Senegal	2012	306,916	111,201	64
Tanzania	2012	1,224,095	389,714	68
Uganda	2011	908,627	850,000	6
Zambia	2010	1,300,000	482,077	63
Zanzibar	2008	200,731	66,497	67
Zimbabwe	2013	622,300	163,922	74
*Total*	*NA*	*9,037,069*	*4,258,495*	*53*

The substantial increase in insecticide cost, firstly caused by increased use of carbamates (2011–2014), followed by replacement with organophosphates (2014–2015), has led to the scaling-down of most PMI-funded IRS programmes. Overall, there was a decrease of 53 % in the total number of structures sprayed between years of peak coverage to 2015 estimates (Table 2). In Malawi, PMI supported the spraying of 97,329 structures in 2010, but the spread of pyrethroid and carbamate resistance resulted in the withdrawal of IRS support in 2012, due to the ‘high cost and short residual action of p-methyl EC’ [27] (Table 2). While the majority of IRS programmes have continued, there is a clear downward trend in coverage from the heights achieved when pyrethroids were the insecticide of choice (Table 2). In Tanzania (mainland), a total of 1,224,095 structures were sprayed in 2012, compared with 389,714 in 2015, with the reduction attributed to ‘the high cost of p-methyl CS’ [39]. Similar sizeable reductions in the number of structures sprayed were reported in Ghana (38 %), Madagascar (56 %), Mali (42 %), and Zambia (63 %). The most commonly cited reasons for the reduced spray coverage were the limited availability of alternative insecticides and increased cost of insecticides, as vector resistance necessitated switching from pyrethroids to organophosphates [40, 41]. PMI-funded IRS was also withdrawn from Angola, Liberia and temporarily in Kenya, although in Kenya political and insecticide registration issues were the main drivers, rather than increased cost of insecticides [21, 42, 43]. In Nigeria, the PMI-funded IRS programme was only intended as a pilot to demonstrate IRS feasibility [44]. It is also important to note that in some countries IRS has been partially funded through other sources, for example in Ethiopia where the Government has taken over spray operations in graduated districts where PMI has developed sufficient capacity [45].

## Evidence for malaria resurgence following withdrawal of IRS

A change in policy from blanket spraying towards focal targeted coverage is a valid, cost-effective strategy in areas of low transmission or at the pre-elimination phase [46, 47]. In the archipelago of Zanzibar, a combination of vector control and improved access to diagnosis and treatment has resulted in a new target of malaria elimination by 2020 [47]. In such a setting the decrease in structures sprayed from a peak of 200,731 in 2008 down to 66,497 in 2015, while maintaining universal LLIN coverage, is perfectly justifiable (Table 2). However, failure to maintain adequate vector control coverage (LLIN and IRS) is likely to result in malaria resurgence to pre-intervention levels. A notorious example of resurgence following withdrawal of IRS is the eradication programme of Zanzibar (1957–1968) which consisted of annual spraying with dieldrin or DDT and had reduced malaria prevalence from 50–60 % to 0–3 % by 1968. In 1979, 11 years after cessation of spraying, malaria had rebounded to close to pre-intervention levels [48].

PMI has ensured that universal coverage with LLINs has been achieved in areas where IRS has been scaled back or withdrawn. Despite this, reducing the number of structures sprayed with IRS is likely to result in an increase in malaria transmission unless complementary control measures in addition to LLINs are implemented. In addition, behavior change communication (BCC) is likely to be important in areas where IRS was previously used and mosquito net use is not yet ingrained [49]. There are already worrying preliminary reports of rapid resurgence following IRS withdrawal.

## Examples

### Tanzania

IRS commenced in 2007 in two districts of Kagera Region, in northwest Tanzania, and was subsequently extended to cover 18 districts. Annual rounds of IRS with the pyrethroid lambdacyhalothrin were conducted between 2007 and 2011 in Muleba District and LLIN distribution was conducted in 2011 targeting universal coverage [39]. National Malaria Indicator Surveys indicated that prevalence of malaria in Kagera decreased substantially from 41 % in 2007–2008 [50] to 8 % in 2011–2012 [51]. A cluster-randomized trial (CRT) was conducted in 2012 to determine whether continued bendiocarb IRS in addition to moderate coverage with LLIN provided any additional benefit. The mean prevalence of *Plasmodium falciparum* in children aged 0.5–14 years, conducted over three time points, was 26.1 % in the arm with LLIN only compared with 13.3 % for bendiocarb IRS + LLIN [12]. IRS spraying was not conducted in Kagera between 2014 and 2016 despite the high malaria prevalence demonstrated in the arm of the CRT where IRS was withdrawn [39].

### Benin

Ouémé department was chosen for IRS due to high prevalence, entomological inoculation rate, and infant mortality [52]. Four rounds of IRS with bendiocarb were conducted in Ouémé, between 2008 and 2011, after which IRS was re-located to the northern department of Atacora [38]. Upon withdrawal of IRS an LLIN distribution campaign for all households in Ouémé took place. The technical rationale for the IRS location change involved the shorter transmission season in the north which can be covered by one round of bendiocarb IRS. Despite 81.8 % of people sleeping under LLIN after the net distribution campaign, entomological indicators of malaria transmission, including higher biting rates, increased significantly in Ouémé after IRS withdrawal [53].

### Uganda

PMI supported IRS in ten northern districts between 2009 and 2014; spraying bendiocarb from 2010 due to an increased frequency of pyrethroid resistance [22]. PMI sentinel sites and Government facilities documented a sustained reduction in malaria cases during this period. Given the success of the IRS campaign in the north, PMI relocated IRS to the eastern region in 2015 (based on National Malaria Control Programme recommendations) where transmission rates were consistently high. In northern districts, where IRS was withdrawn, enhanced case surveillance, robust case management, LLIN distribution targeting universal coverage and BCC to promote net use was intended to sustain the reduced rate of malaria transmission [22]. Despite these measures, in 2015 Uganda reported a sixfold increase in confirmed malaria cases (compared to 2012–2014 average) in northern districts where IRS was withdrawn [54]. This was in contrast to sentinel monitoring in Tororo district, eastern Uganda where a substantial decrease in malaria morbidity was recorded following two rounds of IRS in 2015 [55].

## Conclusions

IRS is a proven strategy for malaria control in a range of endemicity settings and is highly effective for malaria control in sub-Saharan Africa [56]. As a consequence of increased resistance in vector populations to pyrethroids, DDT and carbamates there is critical shortage of insecticides available for IRS use in sub-Saharan Africa (Fig. 2). Resistance to pyrethroids and DDT is particularly widespread, with twelve PMI-funded countries only having carbamate and/or organophosphate insecticides as viable options for IRS (as indicated by WHO cylinder tests) (Fig. 2) [57]. While in Kenya, Malawi, and Benin the situation is even more precarious, with only the organophosphate class of insecticides remaining a viable option for IRS (Fig. 2).

Increasing incipient resistance to bendiocarb, through the G119S mutation in the Ace-1 gene and P450-mediated detoxification, has been reported in several countries [58, 59]. Despite impressive longevity, the high cost of p-methyl CS has resulted in downscaling of most PMI-funded IRS programmes in sub-Saharan Africa. In an attempt to achieve the Millennium Development Goals (MDG) target of 75 % reduction in the global malaria burden by 2015, integrated vector control based on simultaneous spraying of IRS and high coverage with LLIN was implemented concurrently in several countries. WHO recommend that countries investing in combined use of IRS + LLIN should conduct rigorous monitoring and evaluation to determine the degree of additional benefit [60]. While there is varied evidence for enhanced protection, significant additional benefit of IRS + LLIN has been clearly demonstrated in Tanzania and Equatorial Guinea [12, 61]. It is vitally important for more detailed cost-effectiveness analysis to be conducted at the national level in order to determine when and where IRS should be maintained, scaled up, or geographically re-targeted. There is great competition for resources for malaria control and while funding reached $2.7 billion in 2013, this represents a significant shortfall on the estimated $5.1 billion needed annually between 2014 and 2020 [4]. Funding for malaria control has stagnated in recent years with only a 3 % increase in funding between 2012 and 2013 [4]. Inevitably, this leads to competition for resources across various interventions, including new interventions such as seasonal malaria chemoprevention (SMC) in the Sahel sub-region including in Senegal and Mali [62, 63]. LLIN coverage has improved substantially in recent years but usage is still way below the levels expected of successful universal coverage campaigns [64, 65]. In addition, the spread of pyrethroid resistance may necessitate the use of more expensive combination nets with synergists, such as Olyset Plus or Permanet 3.0, which may be more effective in areas of pyrethroid resistance due to raised levels of mixed function oxidases [66, 67]. Unfortunately, with such intense competition for resources IRS is often scaled back to compensate for any shortfalls caused by increased costs of other interventions.

A potential advantage of IRS over other vector control measures, such as LLINs, is that insecticide resistance management is possible through the rotation of different chemical classes. The Global Plan for Insecticide Resistance Management (GPIRM) was developed by the WHO in 2012, but a key factor limiting the implementation of resistance management techniques is the lack of new insecticides with different modes of action [68]. Chlorfenapyr SC formulation of the pyrrole chemical class was evaluated by WHOPES in 2013 and 2014 but was found to have a short residual efficacy of between 0 and 4 weeks [31]. Chlorfenapyr has great potential to be an important IRS insecticide due to the mode of action being different to existing IRS formulations and therefore there is low risk of cross-resistance. However, further development is required to improve the residual performance [69]. Another promising IRS formulation with a different mode of action is the neonicotinoid, clothianidin WP that is currently undergoing laboratory phase WHOPES evaluation [70]. It is critically important that new, long-lasting and affordable insecticides are developed urgently so that a portfolio of viable insecticides are made available for sustainable IRS campaigns with a goal leading towards malaria elimination. The Innovative Vector Control Consortium (IVCC) is likely to have a critical role and is currently in the process of developing new long-lasting IRS insecticides through product development partnerships with industry [71]. In order to encourage a more sustainable market for IRS products UNITAID recently committed $65 million through the IVCC over 4 years which will reduce the price of a new insecticide from $23.50 to $15 by 2020. The rationale is to build up production and batch scale as well as a larger and more competitive market to sustain affordable pricing [72]. Indeed, pyrethroid insecticides were considered expensive when first used for IRS. However, the price decreased from $20 per kg of deltamethrin WP in 1999 to just $4 by 2004 due to several factors including improved manufacture processes, larger scale production and added competition [35, 73].

There have been unprecedented reductions in malaria cases, and all-cause child mortality across much of sub-Saharan Africa in recent years largely as a result of increased spending on vector control, diagnostics and treatment. The subsequent resurgence in malaria cases following the end of GMEP in 1969 highlights the fragility of such gains when control efforts are weakened [74]. If no new cost-effective insecticides are developed there is a real danger that the downward trends for IRS coverage will continue. Malaria resurgence following downscaling of IRS, as reported in parts of Benin, Tanzania and Uganda is a major threat and there are currently no new cost-effective and widely applicable control measures than can feasibly replace IRS for use in combination with LLIN.
